# Mitochondrial Membranes of Human SH-SY5Y Neuroblastoma Cells Express Serotonin 5-HT_7_ Receptor

**DOI:** 10.3390/ijms21249629

**Published:** 2020-12-17

**Authors:** Alessandra Tempio, Mauro Niso, Luna Laera, Lucia Trisolini, Maria Favia, Lucia Ciranna, Domenico Marzulli, Giuseppe Petrosillo, Ciro Leonardo Pierri, Enza Lacivita, Marcello Leopoldo

**Affiliations:** 1 Dipartimento di Scienze Biomediche e Biotecnologiche, Università degli Studi di Catania, via S. Sofia 97, 95123 Catania, Italy; alessandra.tempio@phd.unict.it (A.T.); ciranna@unict.it (L.C.); 2 Biofordrug srl, via Dante 99, 70019 Triggiano (Bari), Italy; 3 Dipartimento di Farmacia-Scienze del Farmaco, Università degli Studi di Bari Aldo Moro, via Orabona 4, 70125 Bari, Italy; mauro.niso@uniba.it (M.N.); mariafavia@hotmail.com (M.F.); 4 Dipartimento di Bioscienze, Biotecnologie e Biofarmaceutica, Università degli Studi di Bari Aldo Moro, via Orabona 4, 70125 Bari, Italy; luna.laera@uniba.it (L.L.); lucia.trisolini@uniba.it (L.T.); ciro.pierri@uniba.it (C.L.P.); 5 Institute of Biomembranes, Bioenergetics and Molecular Biotechnologies (IBIOM), National Research Council (CNR), 70126 Bari, Italy; d.marzulli@ibiom.cnr.it (D.M.); g.petrosillo@ibiom.cnr.it (G.P.)

**Keywords:** serotonin, mitochondria, G protein-coupled receptor, 5-HT7 receptor, cytochrome c oxidase

## Abstract

Mitochondria in neurons contribute to energy supply, the regulation of synaptic transmission, Ca^2+^ homeostasis, neuronal excitability, and stress adaptation. In recent years, several studies have highlighted that the neurotransmitter serotonin (5-HT) plays an important role in mitochondrial biogenesis in cortical neurons, and regulates mitochondrial activity and cellular function in cardiomyocytes. 5-HT exerts its diverse actions by binding to cell surface receptors that are classified into seven distinct families (5-HT1 to 5-HT7). Recently, it was shown that 5-HT3 and 5-HT4 receptors are located on the mitochondrial membrane and participate in the regulation of mitochondrial function. Furthermore, it was observed that activation of brain 5-HT7 receptors rescued mitochondrial dysfunction in female mice from two models of Rett syndrome, a rare neurodevelopmental disorder characterized by severe behavioral and physiological symptoms. Our Western blot analyses performed on cell-lysate and purified mitochondria isolated from neuronal cell line SH-SY5Y showed that 5-HT7 receptors are also expressed into mitochondria. Maximal binding capacity (Bmax) obtained by Scatchard analysis on purified mitochondrial membranes was 0.081 pmol/mg of 5-HT7 receptor protein. Lastly, we evaluated the effect of selective 5-HT7 receptor agonist LP-211 and antagonist (inverse agonist) SB-269970 on mitochondrial respiratory chain (MRC) cytochrome c oxidase activity on mitochondria from SH-SY5Y cells. Our findings provide the first evidence that 5-HT7 receptor is also expressed in mitochondria.

## 1. Introduction

G-protein-coupled receptors (GPCRs) are the largest family of membrane receptors in eukaryotes. About 800 GPCRs have been identified in humans, of which about half have sensory functions, while the remaining half include nonsensory GPCRs that mediate signaling by ligands and are the targets for a majority of drugs in clinical usage [1].

The largest majority of studies focused on GPCRs present on the cell surface and their downstream signaling partners. However, a critical new role is emerging for GPCRs to signal from inside the cell. In fact, intracellular GPCRs were localized in the nuclear membrane, endoplasmic reticulum, lysosomes, and mitochondrial membranes [2].

Recent studies unveiled that various GPCRs are associated with mitochondria. Purinergic receptors were among the first GPCRs to be localized to mitochondria, where they contribute to the regulation of mitochondrial Ca^2+^ uptake [3]. Angiotensin receptors AT1 and AT2 were found in the mitochondria of several cell types. The AT2 receptor was localized on the inner mitochondrial membrane, where its activation results in nitric oxide formation and respiration suppression in various cell types including neurons [4]. Serotonin 5-HT3 and 5-HT4 receptors are present on cardiac mitochondria, where they regulate mitochondrial activities and cellular functions [5]. Melatonin MT1 receptors are present on the outer mitochondrial membrane, where melatonin activates Gαi and blocks adenylate cyclase activity, leading to the inhibition of stress-induced cytochrome c release and caspase activation [6]. Altogether, these studies pose the question as to whether many processes previously thought to be mediated by plasma membrane receptors are also mediated by mitochondrial GPCRs [2].

Serotonin 7 receptor (5-HT7R) is a GPCR broadly expressed in the central nervous system, including the hypothalamus, thalamus, hippocampus, prefrontal cortex, striatum, amygdala, and spinal cord. 5-HT7R controls diverse neural functions such as thermoregulation, the sleep–wake cycle, circadian rhythm, nociception, learning, and memory processing. 5-HT7R dysfunction has been related to neuropsychiatric and neurodevelopmental diseases (depression, anxiety, schizophrenia, epilepsy, impulsivity, and autism spectrum disorder) [7].

5-HT7R is a key component of the molecular cascade involved in the organization and reshaping of neuronal cytoarchitecture during prenatal and postnatal development, as well as in the mature brain. The involvement of 5-HT7R in synaptic plasticity was further demonstrated by studies reporting that its activation rescues long-term potentiation or long-term depression deficits in various rodent models of neurodevelopmental diseases [8]. In fact, the activation of 5-HT7R corrects molecular, electrophysiological, and behavioral alterations in mice models of neurodevelopmental disorders, such as Fragile-X syndrome [9], Rett syndrome, and CDKL5 deficiency disorder [7]. In particular, Valenti and coworkers reported that selective 5-HT7R agonist LP-211 [10] had beneficial effects on the neurobehavioral phenotype of two mouse models of Rett syndrome. Interestingly, the effects were associated with the rescue of mitochondrial abnormalities in the brain [11]. The same group also reported that the reactivation of mitochondrial respiratory chain complexes in the brain of a mouse model of CDKL5 deficiency disorder by treatment with LP-211 rescued the defective brain energy status [12]. This finding was consistent with literature data, as mitochondrial dysfunction and altered mitochondrial dynamics were documented in pathologies characterized by impaired neuronal development [13]. The above studies suggested a direct link between mitochondrial functionality and 5-HT7R, but they did not investigate the mechanism through which 5-HT7R elicited the observed effects. Considering the increasing number of studies reporting the presence of GPCRs on mitochondrial membranes, we searched the literature to find if 5-HT7Rs were ever localized on mitochondrial membrane, but we did not find any evidence for any cell type.

Thus, we addressed the relationship between 5-HT7R and mitochondrial function by investigating the presence of 5-HT7R on the mitochondrial membrane of the SH-SY5Y cell line. Over the last forty years, the SH-SY5Y cell line has been extensively used as a neuronal model due to experimental limitations caused by the inability of primary neurons to propagate in vitro. Consequently, a wealth of biological research has relied on SH-SY5Y cells as a model to investigate central-nervous-system (CNS) disorders, including neurodevelopmental disorders [14]. It is, therefore, not surprising that SH-SY5Y cells have been used to investigate the effects downstream of the activation of 5-HT7R [15,16]. Consistently, Yuksel and coworkers reported 5-HT7R mRNA expression in SH-SY5Y cells [17], even if no study reported the SH-SY5Y cellular expression of a 5-HT7R protein.

Therefore, we investigated the expression of 5-HT7R in the SH-SY5Y cell line, verifying the presence of the receptor at mitochondrial membranes. Then, we determined the total density (Bmax) of 5-HT7R in SH-SY5Y mitochondrial subfraction via Scatchard analysis. Lastly, we estimated mitochondrial respiratory chain (MRC) cytochrome c oxidase (Complex IV) activity in mitochondria extracted from SH-SY5Y before and after incubation with selective 5-HT7R agonist LP-211 or selective antagonist (inverse agonist) SB-269970 through spectrophotometric assays.

## 2. Results

### 2.1. 5-HT7Rs Are Located in Cytosolic and Mitochondrial Fractions of SH-SY5Y Cells

**Figure 1:**
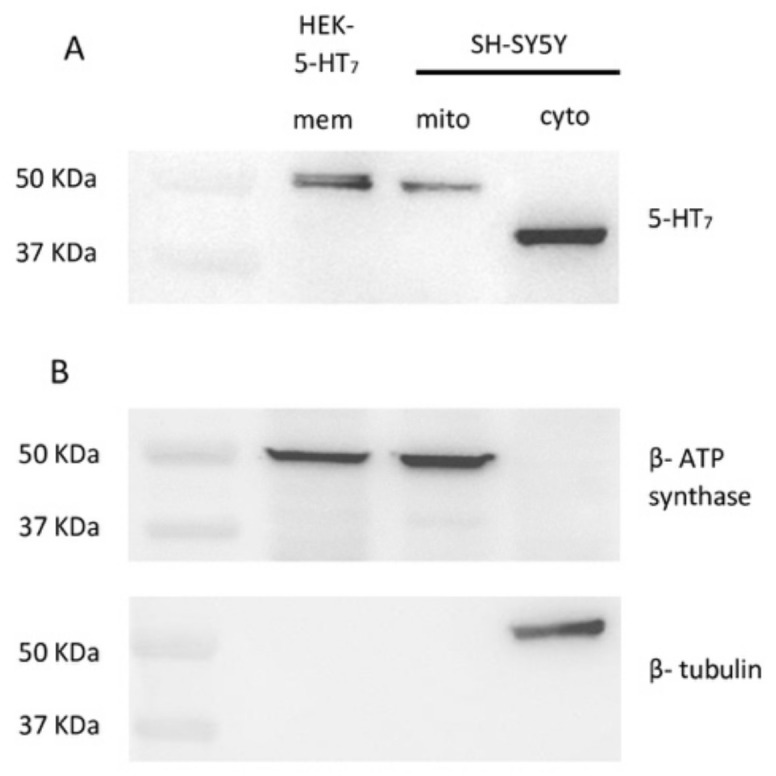
(**A**) Expression of 5-HT7R in cytosolic (cyto) and mitochondrial (mito) enriched fractions obtained from SH-SY5Y cell line. Positive control represented by membranes (mem) obtained from 5-HT7R-stably transfected HEK 293 cells. (**B**) Same fractions of SH-SY5Y analyzed to detect β-ATP synthase (mitochondria marker) and β-tubulin (cytosol marker) expression by sequential reprobing on same blot. Molecular mass markers (KDa) indicated on the left.

We first investigated the expression of 5-HT7Rs in the SH-SY5Y cell line through immunoblotting analysis of cytosolic and mitochondrial-enriched fractions using a rabbit polyclonal antibody against a sequence identical for all human splice variants of 5-HT7R. As a positive control, we used membranes obtained from HEK 293 cells, stably transfected with cDNA for 5-HT7R that express 5-HT7R. These membranes were the very same used in the radioligand binding assay [18]. Western blot analysis revealed that 5-HT7R was present in both cytosolic and mitochondrial fractions (Figure 1A). Two bands were detected at approximately 40 and 50 KDa in the cytosolic and the mitochondrial-enriched fraction, respectively. This data pattern was observed in at least three independent experiments. Results showed that two forms of 5-HT7R are expressed in SH-SY5Y cells.

The expected range was 43–50 KDa and corresponded with the three known 5-HT7R splice variants. 5-HT7R undergoes alternative splicing at the second intron, located in the carboxyl terminus, giving rise to three splice variants in humans (a,b,d) [19]. In addition, 5-HT7R undergoes different post-translational modifications. This receptor contains two consensus sequences for N-linked glycosylation sites in the extracellular N-terminal region [20] and for attachment of saturated fatty acids (i.e., palmitate) to cysteine residues within the protein via thioesterification (S-palmitoylation) [21]. The 40 KDa cytosolic form suggested the presence of a form of the receptor not subjected to post-translational modifications in SH-SY5Y cells [22].

In order to rule out any cross-contamination from the mitochondrial to the cytosolic fraction and vice versa, the same cell fractions were probed using antibodies as marker proteins for specific cellular compartments: an anti-β-ATP synthase antibody for mitochondria, and an anti-β-tubulin antibody for cytosol. Results showed no β ATP synthase band in the cytosolic fraction and no β-tubulin band in the mitochondrial fraction (Figure 1), indicating that there was no contamination in the analyzed fractions.

### 2.2. Saturation-Binding Assay

**Figure 2:**
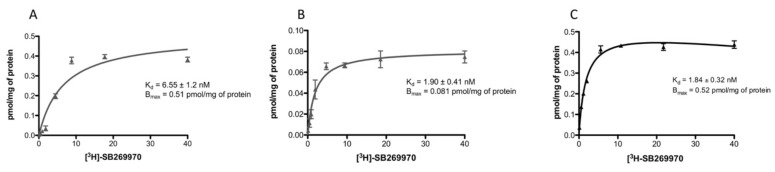
Scatchard analysis with selective 5-HT7R radioligand [^3^H]SB-269970 on (**A**) whole SH-SY5Y cell membranes, (**B**) mitochondrial-enriched fractions obtained from SH-SY5Y cell line, and (**C**) membranes of 5-HT7R-transfected HEK 293 cells.

The presence of 5-HT7R in the SH-SY5Y cell line was investigated with saturation-binding analysis. The assay was performed on both whole SH-SY5Y cell membranes and SH-SY5Y cell mitochondrial fractions. Results demonstrated the presence of 5-HT7R in both preparations, albeit with different expressions. SH-SY5Y cell membrane Bmax was 0.51 pmol/mg of protein (Figure 2A), whereas SH-SY5Y cell mitochondrial fraction Bmax was 0.081 pmol/mg of protein (Figure 2B). Furthermore, experiments gave different Kd values for [^3^H]SB-269970 in whole SH-SY5Y cells (Kd = 6.55 nM) and SH-SY5Y cells mitochondrial-enriched fraction (Kd = 1.90 nM). For comparative purposes, saturation-binding analysis, performed with membranes obtained from HEK 293 cells stably transfected with cDNA for 5-HT7R, is reported in Figure 2C.

Schild regression analysis indicated the presence of a single binding site in the SH-SY5Y cells’ mitochondrial-enriched fraction and the presence of an additional binding site in whole SH-SY5Y cell membranes.

### 2.3. Administration of SB-269970 (but Not LP-211) to Mitochondria Weakly Influences Mitochondrial Respiratory Chain (MRC) Cytochrome c Oxidase Activity

**Figure 3:**
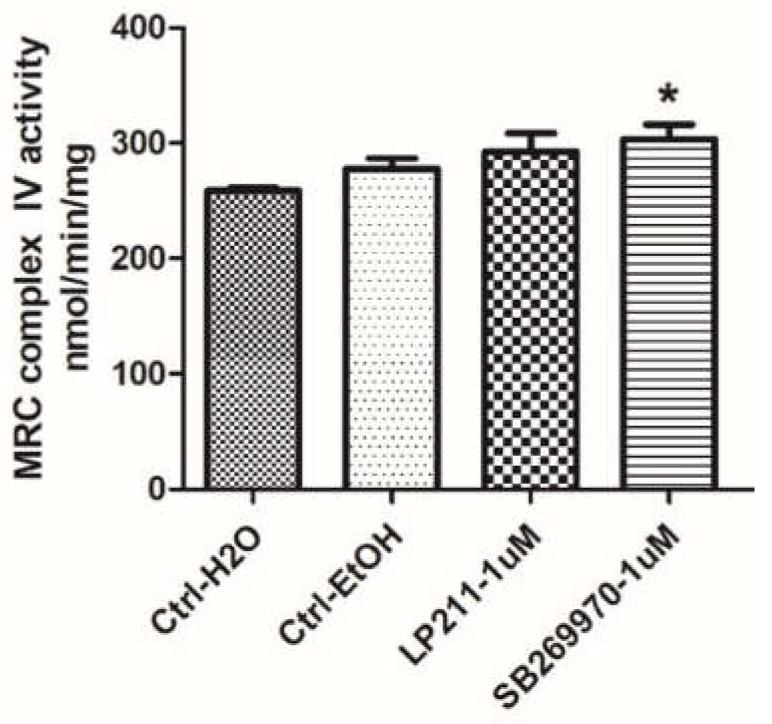
SB-269970 showed a weak stimulating effect on cytochrome c oxidase activity, which was spectrophotometrically measured in mitochondrial fractions from SH-SY5Y cells administered with LP-211 and SB-269970 3 min before measurements. Values represent mean rates (nmol/min/mg) ± SEM obtained from at least four independent experiments. * *p* < 0.05, nonparametric Wilcoxon test between mitochondria administered with SB-269970 and nontreated mitochondria in two controls. Ctrl-EtOH, 10% EtOH in H_2_O.

To investigate whether the mitochondrial function is influenced by the activation of 5-HT7Rs located on mitochondria in the SH-SY5Y cell line, mitochondrial respiratory chain (MRC) cytochrome c oxidase activity was measured in mitochondria purified from SH-SY5Y cells after incubation with selective 5-HT7R agonist LP-211 or 5-HT7R antagonist (inverse agonist) SB-269970 (Figure 3). LP-211 or SB-269970 was dissolved in 10% ethanol in H_2_O. Mitochondria were incubated for 3 min with LP-211 or SB-269970 (1 µM) before the measurements. Cytochrome c oxidase activity was 258.6 ± 4.28 nmol/min/mg in H_2_O and 286.9 ± 29.41 nmol/min/mg in 10% ethanol in H_2_O. Subsequently, the effect of selective 5-HT7R agonist LP-211 on cytochrome c oxidase activity was tested. No statistically significant differences between LP-211 treatment and control were observed. Upon treatment with LP-211, cytochrome c oxidase activity was 292.7 ± 39.51 nmol/min/mg.

Lastly, the effect of selective 5-HT7R antagonist (inverse agonist) SB-269970 on cytochrome c oxidase activity was evaluated. The incubation of mitochondria with SB-269970 resulted in a weak increase in cytochrome c oxidase activity compared to control. Upon treatment with SB-269970, cytochrome c oxidase activity was 303.63 ± 30.48 nmol/min/mg (Figure 3).

## 3. Discussion

5-HT7Rs are expressed in discrete areas of the CNS at the neuronal and astrocyte levels. These receptors are postsynaptically located, and are positively coupled with a Gs or G12 protein [7]. Several studies highlighted the role of 5-HT7R in neuronal plasticity as a key component of the signaling cascade that regulates several processes in various stages of brain development [8]. Studies conducted with selective 5-HT7R agonist LP-211 showed that 5-HT7R activation can correct molecular, electrophysiological, and behavioral defects in various mouse models of neurodevelopmental diseases [7]. Two of the studies showed that 5-HT7R activation is able to reactivate mitochondrial dysfunction in mouse models of Rett syndrome and CDKL5 deficiency [11,12]. The mechanism by which LP-211 had a positive effect on mitochondrial function was not investigated. Serotonin has a role in the biogenesis of mitochondria. In fact, 5-HT2A receptors are responsible for such an effect [23]. There are also studies that provide evidence of the presence of GPCR on mitochondrial membranes, where stimulation of these receptors has an influence on mitochondrial function [2]. This posed the question of whether the observed effect could be mediated by 5-HT7R expressed into mitochondria. From a search of the literature, the presence of 5-HT7R on the mitochondrial membrane has never been investigated. Thus, to address this fascinating issue, we focused on the SH-SY-5Y cell line, which was used to study the cellular effect of 5-HT7R stimulation [15,16]. We first investigated the expression of 5-HT7Rs in the SH-SY5Y cell line through immunoblotting analysis of the cytosolic and the mitochondrial-enriched fractions using a rabbit polyclonal antibody against a sequence identical for all human splice variants of 5-HT7R. Western blot analysis revealed that 5-HT7R was present in both cytosolic and mitochondrial fractions (Figure 1A). Two bands with molecular masses of approximately 40 and 50 KDa were detected, the former present in the cytosolic fraction and the latter in the mitochondrial fraction. The 45–50 KDa range was consistent with the expected molecular mass of 5-HT7R, which has two sites for N-linked glycosylation in the amino terminal region and several sites for phosphorylation. Thus, two protein forms of 5-HT7R could be expressed in SH-SY5Y cells reflecting different levels of glycosylation and/or phosphorylation [22].

Quantification of the 5-HT7R protein was performed by Scatchard analysis in both membranes from whole SH-SY5Y cells and mitochondrial-enriched membranes of SH-SY-5Y cells. We selected radioligand [^3^H]SB-269970 because it shows greater 5-HT7R selectivity compared to that of [^3^H]5-CT and [^3^H]LSD, which are used in routine radioligand binding assays with 5-HT7R-transfected cell lines [24]. 5-HT7R was detected in both preparations at different concentrations. Bmax values were 0.51 pmol/mg of protein (membranes from whole SH-SY-5Y cells) and 0.081 pmol/mg of protein (mitochondrial-enriched membranes of SH-SY-5Y cells). Scatchard analysis agreed with Western blot analysis regarding the expression of 5-HT7R in the mitochondrial membranes of SH-SY5Y cells. The K_d_ value of [^3^H]SB-269970 in the mitochondrial-enriched fraction was 1.9 nM, close to the literature value (K_d_ = 1.7 nM in guinea pig cortex membranes) [25]. In the membranes of whole SY-SH-5Y cells, the K_d_ value of [^3^H]SB-269970 was 6.55 nM, different from the literature data. This difference prompted us to investigate whether the radioligand was interacting with one or more binding sites. Hill plot analysis indicated the presence of a single binding site in the mitochondrial-enriched fraction of SH-SY5Y cells (h = 1.5) and the presence of more than one binding site in membranes of whole SH-SY5Y cells (h = 3.4). Considering that [^3^H]SB-269970 has measurable affinity for 5-HT5a receptor (HT5a Ki = 63.1 nM; 5-HT7 Ki = 1.3 nM) [24], the presence of 5-HT5a receptor protein in membranes of whole SH-SY5Y cells cannot be ruled out.

To our knowledge, this is the first demonstration that 5-HT7R is expressed in the mitochondrial membrane of SH-SY5Y cells.

Once we had detected the presence of 5-HT7Rs on mitochondrial membranes, we tested if 5-HT7R agonist LP-211 or antagonist (inverse agonist) SB-269970 had an effect on the activity of cytochrome c oxidase, which is a critical regulator of oxidative phosphorylation and used as a marker of neural functional activity [26,27]. A recent study showed that stimulation of mitochondrial cannabinoid receptor 1 in a mouse’s hippocampus, coupled with an intramitochondrial Gαi protein, inhibits a soluble adenylyl cyclase, thereby reducing intramitochondrial cAMP levels. This caused a decrease in oxidative phosphorylation system functions and ATP production. These events led to a decrease in brain mitochondrial function required for the acute effects of cannabinoids on synaptic depression and consequent amnesia [28]. Our test showed that 5-HT7R antagonist (inverse agonist) SB-269970 weakly increased cytochrome c oxidase activity, as estimated on mitochondria isolated and purified [29] from the investigated cells. Results indicated that the increase in cAMP caused by LP-211 had no significant effect on cytochrome c oxidase activity. On the other hand, the weak increase in cytochrome c oxidase activity elicited by 5-HT7R inverse agonist SB-269970 might be linked to a reduction in the intramitochondrial levels of cAMP. This might be compatible with findings showing variations of intramitochondrial cAMP levels may upregulate or downregulate cytochrome c oxidase activity [30].

## 4. Materials and Methods

### 4.1. Drugs

SB-269970 ((2R)-1-([3-Hydroxyphenyl]sulfonyl)-2-(2-[4-methyl-1-piperidinyl]ethyl)pyrrolidine hydrochloride—CAS no. 261901-57-9) was purchased by Tocris Bioscience, Bristol, UK. LP-211 (*N*-(4-cyanophenylmethyl)-4-(2-diphenyl)-1-piperazinehexanamide—CAS no. 1052147-86-0) was provided by Enza Lacivita and Marcello Leopoldo.

### 4.2. Cell Culture

SH-SY5Y neuroblastoma cells (cat. CRL-2266, ATCC, LGC Standards, Sesto San Giovanni, Italy) were cultured in a 1:1 mixture of Eagle’s Minimum Essential Medium (cat. 15-010-CVR, Corning, SIAL, Roma, Italy) and Ham’s F12 Medium (cat. 10-080-CVR, Corning). This medium was supplemented with 10% (v/v) heat-inactivated fetal bovine serum (cat. 35-079-CV, Corning), 1% (v/v) glutamine (cat. ECB3000D, Euro Clone, Pero, Italy) and 1% (v/v) penicillin–streptomycin (cat.30-002-CI, Corning). Cells were cultured in T75 flasks at 37 °C with 5% CO_2_ at saturated humidity and kept below 25 passage to avoid senescence.

### 4.3. Mitochondrial-Enriched Fraction

Cells grown in T75 flasks were detached and centrifuged at 125 *g* for 5 min, the supernatant was discarded, and cells were resuspended in Ringer NaCl buffer (135 mM NaCl, 20 mM HEPES, 0.8 mM MgSO_4_, 3 mM KCl, 1.8 mM CaCl_2_, 11 mM D-glucose, pH = 7.5) [31]. Afterward, cells were centrifuged at 125 *g* for 5 min, suspended in 2 mL of A buffer (sucrose 320 mM, Tris-HCl 5 mM, EGTA 2 mM, pH = 7.4), and homogenized with a glass–Teflon grinder kept in ice. The homogenate was centrifuged at 4 °C for 6 min at 2000 *g* to remove nuclei and tissue particles, while the supernatant was collected and centrifuged at 4 °C for 15 min at 12,000 *g* to pellet mitochondria. Lastly, the pellet was washed with a buffer in order to reduce the cytosolic contamination.

### 4.4. Western Blot Analysis

The mitochondrial-enriched fraction, as described above, was obtained, and treated with RIPA buffer (cat. R0278, Sigma Aldrich, SIAL, Roma, Italy) and protease inhibitor cocktail (cat. P8340, Sigma Aldrich). The mitochondrial lysate was centrifuged at 4 °C for 15 min at 12,000 *g*, and protein concentration in the supernatant was dosed with DC Protein Assay (cat. 500111, Bio-Rad, Bio-Rad Laboratories, Segrate, Italy). Denatured proteins were separated through SDS-PAGE using Mini Protean TGX Stain-Free gels at 10% polyacrylamide (cat. 456-8034, Bio-Rad) and transferred in a 0.2 um PVDF membrane (cat. 1704156, Bio-Rad) using Trans Turbo Blot Transfer System. Membranes were blocked with 5% nonfat milk in TBS-Tween 20 0.1% for 1 h at room temperature and incubated overnight with an anti-5-HT7 (cat. IMG-368, dilution 1:125, Imgenex, Bio-TECHNE, Milano, Italy), anti-β-tubulin (cat. T8328, Sigma Aldrich, dilution 1:5000) and anti-β-ATP synthase (cat. MABS1304, EMD Millipore, dilution 1:1000) antibodies. Membranes were rinsed three times in TBS-Tween 20 0.1% and incubated with either antimouse (cat. G-21040, dilution 1:2000, ThermoFisher Scientific, Life Technologies Italia, Monza, Italy) or antirabbit (cat. AP307P, dilution 1:2000, EMD Millipore, Sigma Aldrich) antibody. Blots were revealed using Clarity Western ECL Substrate (cat. 170-5060, Bio-Rad) through UVITEC Cambridge Chemiluminescence Imaging System.

### 4.5. Cytochrome c Oxidase Activity Measurements

To estimate cytochrome c oxidase (Complex IV) activity, we performed spectrophotometric assays with or without mitochondrial treatment with selective 5-HT7R agonist LP-211 (1 µM) or 5-HT7R antagonist SB-269970 (1 µM), using a standard method [29] with some modifications. LP-211 or SB-269970 was dissolved in 10% ethanol in H_2_O because of low solubility in pure H_2_O. To evaluate the effect of ethanol in the medium, the activity of cytochrome c oxidase was measured by incubating mitochondria for 3 min using H_2_O or 10% ethanol in H_2_O. No significant difference in cytochrome c oxidase activity was observed between the two tests. Mitochondria, obtained as described above, were subjected to three cycles of freeze and thaw in hypotonic potassium phosphate buffer (20 mM, pH = 7.4) to maximize the enzymatic rates. Then, 50 µg of mitochondria was incubated for 3 min with LP-211 (1 µM) or SB-269970 (1 µM) in 10% ethanol in H_2_O or the medium alone, as the control condition in a solution composed by 250 µL of potassium phosphate buffer (0.1 M, pH = 7.5), 5 µL of n-dodecyl-β-D-maltoside (150 mM), and H_2_O to reach the volume of 950 µL in cuvette. The reaction began by adding 50 μL of reduced cytochrome c (1 mM). The decrease in absorbance at λ = 550 nm due to the oxidation of cytochrome c was monitored. Cytochrome c oxidase specific activity was checked by adding 20 µL of KCN 60 mM.

### 4.6. SH-SY5Y Membrane Preparation for Saturation-Binding Assay

The membrane preparation was carried out as described by Colabufo et al. with minor modifications [32]. Briefly, SH-SY5Y cells were cultured to 80% confluence; then, the medium was removed, and cells were rinsed in PBS. After detaching, cells were suspended in ice-cold 10 mM Tris-HCl (pH 7.4), containing 0.32 M sucrose and homogenized in a Potter-Elvehjem homogenizer (Teflon pestle). The homogenate was centrifuged at 31,000 *g* for 15 min at 4 °C, and the supernatant was discarded. The final pellet was resuspended in ice-cold 10 mM Tris-HCl (pH 7.4) and stored at −80 °C until use.

### 4.7. Saturation-Binding Assay

Saturation experiments were carried out as previously reported with minor modification [18]. 5-HT7Rs were radiolabeled using [^3^H]-SB269970 (PerkinElmer Life and Analytical Sciences, Boston, MA, USA) at concentrations in the range of 0.1–20 nM. Samples containing 100 µg of SH-SY5Y cells membranes or 70 µg of SH-SY5Y cells mitochondrial-enriched fraction, radioligand, and 10 µM SB-269970 (Tocris Bioscience, Bristol, UK) to determine nonspecific binding were incubated in a final volume of 0.5 mL (50 mM Tris-HCl, pH 7.4, 4 mM MgCl_2_, 0.1% ascorbic acid, 10 µM pargyline hydrochloride) for 20 min at 37 °C. The suspension was filtered through a Whatman GF/C glass microfiber filter (presoaked in 0.3% polyethylenimine for at least 20 min prior to use). Filters were washed 3 times with 1 mL of ice-cold buffer (50 mM Tris-HCl, pH 7.4). Scatchard parameters (K_d_ and B_max_) and Hill slope (n_H_) were determined by nonlinear curve fitting, using Prism version 5.0 GraphPad software.

### 4.8. Statistical Analysis

Scatchard analysis data were analyzed by applying one-way repeated-measures analysis of variance (ANOVA test), and unpaired t test followed as a post hoc test. Results were reported as mean ± SEM (standard error of the mean) of at least two to three independent experiments, performed in triplicate. Statistical significance was accepted at *p* < 0.05. Similarly, cytochrome c oxidase activity data represent mean rates (nmol/min/mg) ± SEM obtained from at least four independent experiments. *, *p* < 0.05, nonparametric Wilcoxon test between mitochondria administered with LP-211 and SB-269970, and nontreated mitochondria.

## 5. Conclusions

The data presented here are the first evidence that 5-HT7R is expressed in mitochondria on the human neuroblastoma SH-SY5Y cell line. These results are of great relevance in future studies to investigate the expression and functional role of 5-HT7R on the mitochondria of primary neuronal cultures. These aspects are particularly fascinating considering the role of 5-HT7R in neural circuit development and structural plasticity [33], and the role of mitochondria in synaptic transmission [34] in physiological conditions and pathologies characterized by mitochondrial dysfunction, such as Alzheimer’s disease, Parkinson’s disease, and Fragile X syndrome.

## Abbreviations

5-HT	5-hydroxytryptamine
cAMP	cyclic Adenosine MonoPhosphate
CNS	Central Nervous System
GPCR	G-protein-coupled receptor
MCR	Mitochondrial respiratory chain
